# Efficacy of Peripheral Nerve Stimulator Guided Pectoral Nerve Block-1 and Serratus Anterior Plane Block for Post-operative Analgesia in Modified Radical Mastectomy: A Randomized Controlled Study

**DOI:** 10.7759/cureus.56258

**Published:** 2024-03-16

**Authors:** Devyani J Desai, Velmayil Murugesan Ananda Jyothi, Ruchi Pathak

**Affiliations:** 1 Anesthesiology, Medical College Baroda, Vadodara, IND

**Keywords:** postoperative pain, bupivacaine, peripheral nerve stimulator, serratus anterior muscle, pectoral nerve, nerve block, modified radical mastectomy, breast carcinoma

## Abstract

Background: Breast carcinoma is one of the most common cancers in present-day women worldwide, hence surgical intervention for the same is inevitable. General anesthesia being the preferred technique, the selection of appropriate postoperative pain management is a major concern in which superficial fascial plane chest wall blocks play a pivotal role. We aimed to prove the efficacy of peripheral nerve stimulator-guided pectoral nerve-1 (PEC 1) block and serratus anterior plane (SAP) block for postoperative analgesia in modified radical mastectomy.

Methods: This prospective randomized controlled clinical study comprised 60 females undergoing modified radical mastectomy and was randomly allocated to two groups. Group A patients received general anesthesia while, in addition to general anesthesia, group B patients received PEC 1 and SAP blocks. Postoperatively the active and passive visual analog score (VAS), duration of analgesia, cumulative requirement of rescue analgesics in the first 24 hours and associated perioperative complications were noted. All quantitative data were analyzed by student t-test and qualitative data by chi-square test using MedCalc software 12.5.

Results: VAS score for first 24 hours in group B was lower at rest, on pressure over the surgical site as well as on movements compared with the patients in group A with the p-value being < 0.0001 at all time intervals. Time for receiving first rescue analgesia was shorter (1.25±0.56hour vs 20.05±7.78hour, p<0.001) with the significantly higher requirement of cumulative doses of tramadol in the first 24 hours in patients belonging to group A (233.33±47.95mg vs 110±31.62 mg, p<0.001).

Conclusion: PEC 1 and SAP blocks given under peripheral nerve stimulator guidance have a high success rate and are reliable in providing adequate postoperative analgesia for patients undergoing modified radical mastectomy.

## Introduction

Breast cancer is one of the most common cancers among modern-day women worldwide and the second most common in India [1-3]. Despite the neuroendocrine, cytokine, and metabolic responses affecting the immune system by carcinoma, surgery remains the mainstay while managing breast carcinoma [4]. The type of surgery, anesthesia, and perioperative pain management play a pivotal role in managing patients with breast carcinoma. hence managing such cases is a great challenge for anesthesiologists [4]. Inadequate analgesia may affect the respiratory mechanics during the immediate postoperative period, poor wound healing, and present as chronic pain, and insomnia with psychological abnormalities [4].

General anesthesia is the most used mode of anesthesia in case of breast surgeries and perioperative pain management is multimodal including opioids and/or regional anesthetic techniques. The thoracic epidural and thoracic paravertebral blocks were the gold standard regional anesthesia techniques, as they provide excellent postoperative analgesia but have their limitations including failure, requiring expertise as there are angulations of spinous process at the thoracic vertebral level leading to multiple punctures, formation of hematoma and sympathetic blockade leading to hypotension and bradycardia that may go unnoticed during the postoperative period in the ward [5,6].

In 2011, Blanco et al. described the PEC blocks (PEC 1 and 2), which involved depositing the local anesthetics at the interfascial plane between the pectoralis major and minor muscles [2]. Later in 2013, they modified the PEC 2 block as the serratus anterior plane (SAP) block which covers the innervation of the whole breast area along with the axilla [2,3]. The safety and reliability of these blocks when given under the guidance of ultrasound have been reported by the prior literature with the requirement of only a minimal dose of opioid perioperatively with the absence of sympathetic blockade ultimately leading to decreased incidences of postoperative nausea-vomiting, and pulmonary complications, unlike opioids which cause respiratory depression and decreased post-anesthetic care unit stay [2-4]. However, these blocks are superficial and can be given under peripheral nerve stimulator (PNS) guidance as both blocks involve the fascial plane covering muscles. Secondly, many centers have issues regarding ultrasound like availability, adequate learning curve, and expertise. Also, literature regarding combining these two blocks for MRM surgery is sparse [7]. Hence, we decided to study these blocks under PNS guidance being advantageous with easy availability for an anesthesiologist in patients undergoing modified radical mastectomy (MRM) with the primary objective of comparing active and passive visual analog scores (VAS) between two groups. We hypothesized the provision of better postoperative analgesia with the addition of PEC 1 and SAP blocks in comparison to systemic drugs after MRM. The secondary objectives were the duration of postoperative analgesia, the cumulative requirement of rescue analgesic in first 24 hours postoperatively and associated perioperative complications.

## Materials and methods

This prospective randomized study was conducted at a tertiary care center for the duration from September 2019 to October 2020, after seeking permission from the Institutional Ethical Committee for Human Research-Post Graduate Research, Medical College and SSG Hospital, Baroda (IECHR-PGR/102-19) and registering in the Clinical Trials Registry-India (CTRI/2019/11/022086). The study was conducted in adherence to the Consolidated Standard of Reporting Trials (CONSORT) guidelines.

We recruited 60 females from the American Society of Anesthesiologists (ASA) grade I and II having unilateral breast carcinoma scheduled to undergo MRM and were able to understand the study protocol. The patients having an allergic history to study drugs, locally advanced breast malignancies with skin ulcerations or any infiltration of the chest wall, bleeding disorders or on anticoagulants/long-term analgesic therapy, severe cardiac, renal, hepatic, respiratory disease and previous history of neurological/ neuromuscular disorders were excluded. After dividing all patients into two groups randomly using computer-generated random numbers, (www.randomizer.org) allotment was done to one of the groups, namely group A in which patients were given conventional general anesthesia with systemic analgesics and group B in which patients were given general anesthesia along with PEC 1 and SAP blocks performed.

All patients undergo pre-anesthetic check-ups with thorough airway assessment and local site examination in concern with block a day before surgery. Written informed consent was taken and adherence to standard protocol for nil per oral status was done. All the patients under study were explained about the procedure, risks, and usage of data for research and educational purposes. On the day of surgery inside the operation theatre, an intravenous line was secured with an 18 G cannula in the contralateral hand and baseline vitals were recorded using ASA standard monitors. Five minutes before induction, all patients were premedicated with intravenous injections of fentanyl 2mcg/kg, dexamethasone 1 mg/kg, and glycopyrrolate 0.2mg. After pre-oxygenation, anesthetic induction was done with intravenous propofol 2mg/kg and achieving adequate relaxation with succinylcholine 2mg/kg, tracheal intubation was performed and confirmed by square wave capnometry. Intraoperatively, anesthesia was maintained according to standard protocol with sevoflurane and vecuronium bromide while group B received vecuronium bromide after performing blocks.

All blocks were performed in patients positioned supine with slight abduction of the ipsilateral arm. A total drug of 30mL, 0.25% bupivacaine was prepared for nerve block. Under all sterile precautions, a 22 gauge, 50mm insulated nerve stimulator needle (Locoplex, Vygon) with a syringe filled with local anesthetics attached to the extension tubing was inserted perpendicular to the skin keeping the landmark as the point of intersection of the angle of Louis (second rib) and the anterior axillary line. PNS was set initially at 2.5-3mA, 0.1ms and 1Hz, the needle was slowly advanced till the pectoralis muscle contractions were achieved. On the persistence of muscle contraction to 0.6mA of current, 10mL of 0.25% bupivacaine was slowly injected in increments with frequent negative aspirations. The SAP block was given at the point of intersection of the fifth rib and the mid-axillary line. The needle was inserted perpendicular to the skin with PNS calibrated as 2.5-3 mA, 0.1 ms and 1Hz and the needle was slowly advanced till the serratus anterior muscle contractions were noted and then gradually reduced to 0.6 mA. After confirming the persistence of muscle contractions, 20 mL of 0.25% bupivacaine was injected as above.

Intraoperatively, fentanyl 0.25mcg/kg was used as an analgesic during the maintenance phase if there was a 20% increase in baseline vitals. Toward the end of the surgery, all patients received paracetamol 1g intravenously in both groups and were extubated after achieving the criteria for extubation.

Postoperatively, the patient has been enquired regarding the intensity of pain at rest, with mild pressure over the surgical site and with movements over the shoulder joint. The VAS score was assessed immediately after extubating the patient, after 30 minutes, then hourly for the first six hours, two hourly for the next six hours and at 18 and 24 hours. On attaining the VAS score ≥4, patients received the rescue analgesia in the form of intravenous tramadol 2 mg/kg and the time at which first rescue analgesia was needed and the total number of doses given in first 24 hours postoperatively, were noted. The time from when the blocks were given till the supplementation of first rescue analgesia was considered as the duration of postoperative analgesia. Perioperative complications like pneumothorax, local anesthetic systemic toxicity, and postoperative nausea/vomiting (PONV) were noted and managed accordingly.

The pilot study done on 10 patients who underwent MRM in the same institute was used to generate the data and calculate the sample size. The VAS on movements at 12 hours postoperatively was 4.4±0.65 vs 3.2±0.6. Adding the standard effect size 2 and assuming a two-sided alpha error of 0.01 and a beta error of 0.2, the sample size required was 52. Considering the dropouts, the sample size was increased to 60 (30/group) patients. The Excel sheet was prepared from observed data and statistical analyses were performed using MedCalc (version 12.5.0, Ostend, Belgium). For all continuous variables, results were presented as mean±standard deviation (SD) categorical variables as numbers or percentages. The student “t” test was applied to see the statistical significance of continuous data like VAS, the cumulative dosage of analgesic in the first 24 hours, and the duration of postoperative analgesia between the two groups. Pearson's chi-square test was used to obtain the association between categorical variables like ASA grading, and peri-operative complications. The significance of statistical analysis was judged by the p-value and p<0.05 was considered significant.

## Results

**Figure 1:**
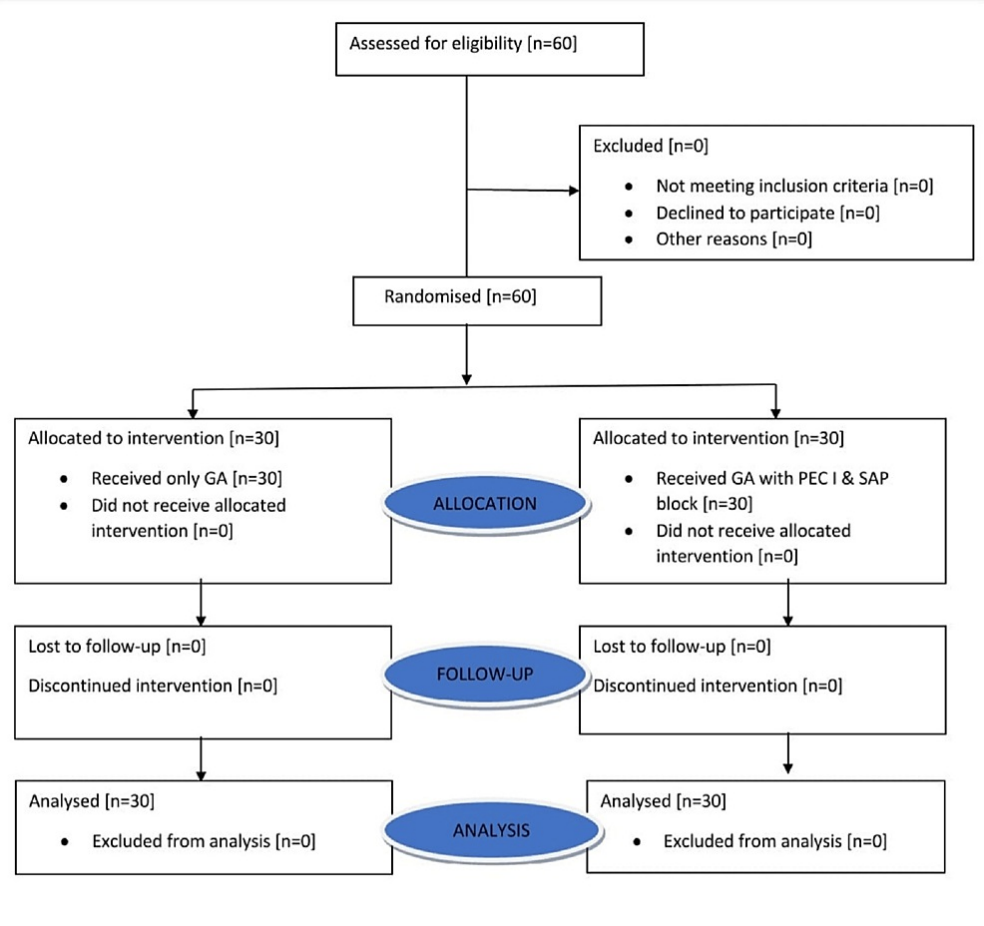
Consolidated Standards of Reporting Trials (CONSORT) flow diagram

In this study, the patients were followed and analyzed as depicted in the flow diagram (Figure 1).

The demographics were comparable in both groups (Table 1).

**Table 1:** Demographic profile Values are presented as mean±standard deviation or numbers, Statistical analysis: Student t-test except ASA grading by Pearson’s Chi-square test Abbreviations: kg - kilogram, cm - centimeter, ASA - American Society of Anesthesiologists

Parameter	Group A	Group B	P-value
Age (years)	50.2±13.1	44±12.52	0.06
Weight (kg)	54.46±5.33	52.84±6.67	0.30
Height (cm)	154.31± 3.47	154.28 ± 3.51	0.97
ASA Grading I/II	18:12	15:15	0.44
Duration of Surgery (minutes)	124.40±11.86	128.60±10.40	0.15

**Figure 2:**
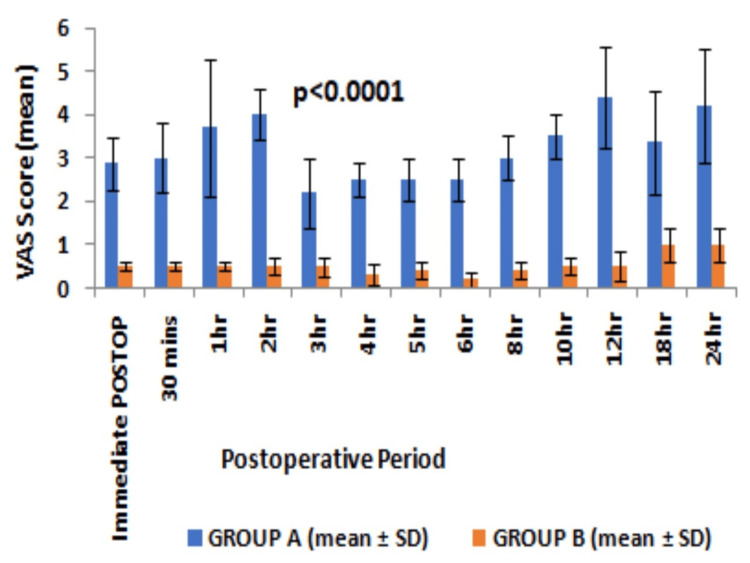
Postoperative VAS score (mean and SD) observed at different time intervals at rest VAS - visual analog score, SD - standard deviation, Statistical analysis - Student “t” test

Comparing both the groups, the VAS score at rest was <4 at all time intervals till 24 hours postoperatively in group B while the VAS score was observed >4 in first two hours post-surgery in group A requiring analgesia much earlier (Figure 2).

**Figure 3:**
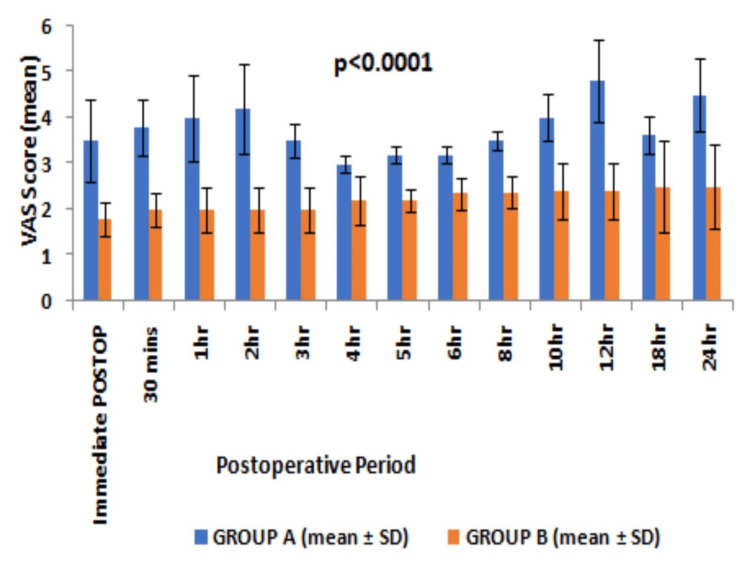
Postoperative VAS score (mean and SD) observed at different time intervals with pressure over the surgical site VAS - visual analogue score, SD - standard deviation, Statistical analysis - Student “t” test

**Figure 4:**
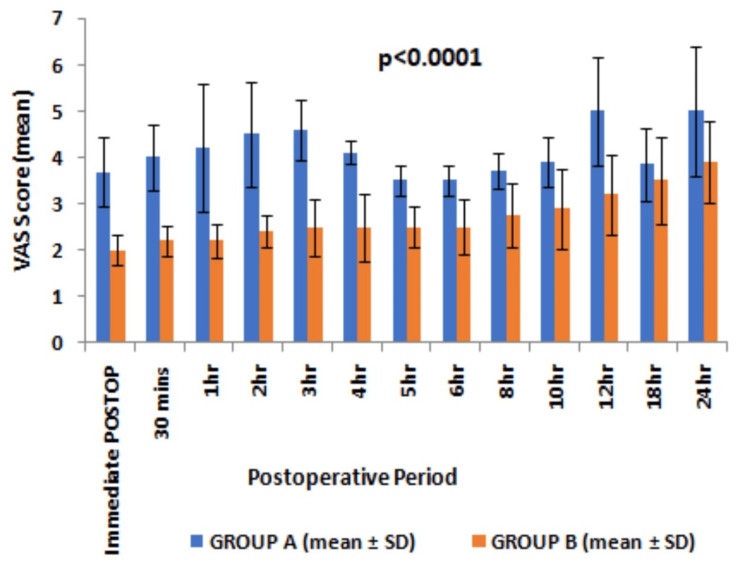
Postoperative VAS score (mean and SD) observed at different time intervals on movements P-value was <0.0001 for every hour depicted here except at the 18th hour - p=0.01 and at the 24th hour p=0.0004. VAS - visual analogue score, SD - standard deviation, Statistical analysis - Student “t” test

The VAS score remained higher than the rest but never >4 on the application of pressure over the surgical site and on movements in patients with group B till 24 hours postoperatively (Figures 3, 4).

Moreover, the VAS score remained significantly lower at any point time during first 24 hours of a postoperative period in group B as compared to group A. The patients in group A experienced significantly higher VAS even at rest than group B patients, thereby requiring rescue analgesia of a minimum of two doses during first 24 hours. The time to first rescue analgesia was significantly higher suggesting a longer duration of postoperative analgesia in group B than in group A (20.05±7.78 vs 1.25±0.56, p<0.0001, 95% CI 15.95 to 21.65, degree of freedom 58) (Table 2). The cumulative doses of intravenous tramadol required in first 24 hours after surgery were higher in group A than in group B (233.33±47.9 vs 110±31.62, p<0.001, 95% CI -144.3 to -102.34, degree of freedom 58) (Table 2). No significant complications were observed except PONV in 10% of cases in group A (Table 2).

**Table 2:** Comparison of study prameters Values are presented as mean±standard deviation or numbers, Statistical analysis: Student t-test except peri-operative complications by Pearson’s Chi-square test Abbreviations: mg - milligram, hr - hour, PONV - postoperative nausea/vomiting

Parameter	Group A	Group B	Mean difference	95% CI	DF	P-value
Duration of postoperative analgesia (hr)	1.25±0.56	20.05±7.78	18.8	15.949, 21.651	58	<0.001
The cumulative dose of tramadol required in the first 24hours postoperatively[mg]	233.33±47.95	110±31.62	-123.33	-144.3, -102.339	58	<0.001
PONV	10%	0	10.0%	-4%, 26.5%	1	0.2361
Pneumothorax	0	0	-			
Local Anesthetic Systemic Toxicity	0	0				

## Discussion

The major concern after surgery like MRM is the provision of adequate analgesia, early ambulation, avoiding any cardiac or respiratory complications leading to early discharge, and reducing postoperative morbidity and mortality.

Our study showed effective postoperative analgesia with less requirement of analgesic supplementation in patients with the addition of PNS-guided PEC 1 and SAP blocks in MRM patients as assessed with active and passive VAS scores in the first 24 hours of the postoperative period compared to the conventional systemic analgesics. Most patients required a dose of rescue analgesia in the first 24 hours during the second hour, 12th hour, and 23rd hour postoperatively in group A. 

Chest wall blocks under ultrasound guidance were introduced by Blanco et al. in 2011 with PEC 1 and 2 and modified PEC 2 block, i.e., SAP block involving thoracic-intercostal nerves covering T2-T9 in 2013 for hemithorax analgesia [2,3]. Using the continuous catheter technique with PEC block, he found occasional requirements of opioid analgesia in the postoperative period [2]. Kumar et al. also observed a significant reduction in postoperative VAS score at rest and on abduction with less tramadol consumption in 24 hours after using PECs block under ultrasound guidance in patients after MRM [4]. Similarly, Thomas et al. observed reduced postoperative analgesic requirement and pain scores after infiltration at the SAP and between pectoralis major and minor muscles done under direct vision [8]. The study done by Najeeb et al. included combining these two blocks with general anesthesia in patients posted for MRM surgeries to have significantly better postoperative pain control in comparison to general anesthesia alone [7]. Chandni et al. observed higher efficacy of PEC 2 block when compared to erector spinae plane block in patients of MRM in terms of postoperative analgesia and opioid consumption [9]. Eldeen observed a longer duration of pain-free period after PECs block in comparison to thoracic spinal blockade in patients posted for conservative breast surgeries [10]. The modified radical mastectomy traumatizes a larger area of the breast where this novel interfascial block technique would be helpful as it targets to cover innervations of the whole breast area including the axilla. The PEC1 block aims to involve the medial pectoral nerve and lateral pectoral nerve which carry the nociceptive and proprioceptive fibers [2]. The lateral pectoral nerve travels between the pectoralis major and pectoralis minor muscle. It supplies the pectoralis major muscle while, the medial pectoral nerve innervates both, the pectoralis major and minor muscles [2,10]. The long thoracic nerve, thoracic-intercostal nerves from T2-T9, and thoracodorsal nerve (nerve to latissimus dorsi) innervating the axillary, medial, and lateral mammary area with supply to serratus anterior and latissimus dorsi muscles can be targeted by using the SAP block [3,11]. We preferred to use PNS as it is easily available, simple to perform, reliable, and effective.

As we wanted a long duration of action with predominant sensory effects, we used 0.25% bupivacaine. The volume used in our study was 30 mL of which 10 mL was used in PEC 1 and 20 mL in SAP block which aligns with the other studies except the study done by Najeeb et al. who used 20 mL in PEC block [4,7,8].

Successful demonstration of the analgesic effect of conventional thoracic epidural in MRM patients done by various authors. However, the disadvantages included hypotension, post-dural puncture headache, unavailability of syringe pumps in general wards, and complications of under/overdosage of the drug used for epidural infusions [6,8]. Paravertebral blocks were also used successfully by many authors. However, the optimal technique whether single, multiple, or continuous to establish a sufficient and reliable paravertebral block for breast surgery has not been clarified yet [5,12]. The possibility of sparing of pectoral nerves with inadequate analgesia of the chest wall and increased risk of pneumothorax requiring post-operative monitoring was found to be associated with multiple injection techniques which is suggested for an effective paravertebral block making it unsuitable for the day care procedure [13].

Except for 10% of patients in group A had PONV, no other complications like pneumothorax or local anesthetic toxicity were observed in both groups. Similarly, Kumar et al. and Chandni et al. did not observe any complications using the PECs block [4,9]. Morioka et al. observed reduced incidences of PONV in patients who received PECs block in addition to general anesthesia [14]. The risk of local anesthetic toxicity remains low with the SAP block as compared to other fascial plane blocks due to the requirement of lower volume for a higher spread and the lower rate of absorption as the plane is avascular [15]. This can be considered an advantage over alternative techniques such as intercostal nerve block.

The limitation of our study being single-blinded is the observer bias cannot be ruled out. To reduce the bias, only a single anesthesiologist performed all the blocks. We used single concentration and single local anesthetic without adjuvants, so more research might be required in the future to study the duration of analgesic effect with different drugs, concentrations, and adjuvants which also might be helpful in the prevention of chronic pain after MRM. The study results also cannot imply upon another group of patients like obese where the large breast tissue may require to be handled by another person for needling and ultrasound technique might be more accurate enough to see the needle entry and drug deposition at the right fascial plane.

## Conclusions

The PNS-guided PEC 1 and SAP blocks are effective and reliable in providing postoperative analgesia with less consumption of opioids in patients undergoing MRM. These blocks are superficial and can be easily employed under the PNS guidance. This may enhance its application on a large scale in the absence of ultrasound.
